# Workshop report: the clinical application of data from multiplex assays of variant effect (MAVEs), 12 July 2023

**DOI:** 10.1038/s41431-024-01566-2

**Published:** 2024-03-04

**Authors:** Sophie Allen, Alice Garrett, Lara Muffley, Shawn Fayer, Julia Foreman, David J. Adams, Matthew Hurles, Alan F. Rubin, Frederick P. Roth, Lea M. Starita, Leslie G. Biesecker, Clare Turnbull

**Affiliations:** 1 Division of Genetics and Epidemiology, The Institute of Cancer Research, London, UK; 2 St George’s University Hospitals NHS Foundation Trust, Tooting, London, UK; 3 Department of Genome Sciences, University of Washington, Seattle, WA USA; 4 Brotman Baty Institute for Precision Medicine, Seattle, WA USA; 5 European Molecular Biology Laboratory, European Bioinformatics Institute, Wellcome Genome Campus, Hinxton, Cambridge, UK; 6 Wellcome Sanger Institute, Wellcome Genome Campus, Hinxton, Cambridge, UK; 7 Bioinformatics Division, The Walter and Eliza Hall Institute of Medical Research, Parkville, VIC Australia; 8 Department of Medical Biology, University of Melbourne, Melbourne, VIC Australia; 9 Department of Computational and Systems Biology, University of Pittsburgh School of Medicine, Pittsburgh, PA USA; 10 Donnelly Centre and Departments of Molecular Genetics, University of Toronto, Toronto, ON Canada; 11 Lunenfeld-Tanenbaum Research Institute, Sinai Health, Toronto, ON Canada; 12 Center for Precision Health Research, National Human Genome Research Institute, National Institutes of Health, Bethesda, MD USA; 13 The Royal Marsden NHS Foundation Trust, London, UK

**Keywords:** Genetic testing, Cancer genetics, Data publication and archiving

## Abstract

Clinical classification of genomic variants identified on sequencing is often challenging, with many variants classified as Variants of Uncertain Significance (VUS) on account of insufficient evidence. Advances in sequencing and gene synthesis has made feasible multiplexed assays of variant effect (MAVEs), which quantify the functional impact of many thousands of genomic variants in a single experiment. These assays and the functional evidence they generate have the potential to empower more accurate clinical variant classification. However, there are many outstanding challenges and opportunities that require joint resolution and specification, thus necessitating communication between the research scientists who have designed and performed MAVEs and the clinicians and diagnostic scientists who will apply their data to clinical variant classification. In the ‘Clinical Application of MAVE Data’ workshop, held on 12th July 2023 at the Wellcome Connecting Science Conference Centre in between two relevant research meetings, ‘Curating the Clinical Genome 2023’ and the ‘Mutational Scanning Symposium 2023’, 44 key scientific and/or clinical stakeholders were brought together to consider important questions relating to clinical application of MAVE data, such as quantitative validation, variant truth-sets, platforms and standards for dissemination of MAVE data. The outcomes and possible next steps that were discussed encompassed development of focused workshops to develop consensus recommendations, creating a MAVE evaluation working group, and collaboration of ClinVar and MaveDB to enact software changes that support enhanced functional data submission.

## Introduction

The American College of Medical Genetics and Genomics (ACMG) and Association for Molecular Pathology (AMP) published a set of standards in 2015, hereafter termed the ACMG framework, which separated the evidence used in clinical variant classification across 28 criteria, weighted with different evidence strengths [1]. Since then, some of these criteria have been evolved and further specified, providing more explicit methodology for evidence quantitation. Under the auspices of the Sequence Variant Interpretation (SVI) group of the ClinGen consortium, a methodology was published in 2019 by Brnich et al. (hereafter termed Brnich-SVI validation approach), for clinical validation of assay data [2]. This clinical validation comprises assessment and quantitation of an evidence strength for functional assays, which is based on the concordance of assay results with ‘variant truth-sets’, comprising variants formally classified as pathogenic or benign using a clinical framework and involving orthogonal (i.e., non-functional) data sources.

Whilst functional assays have historically analysed only small numbers of variants due to low throughput technologies, recent technology advances have allowed development of multiplex assays of variant effect (MAVEs), through which the effect of many (potentially all possible) single nucleotide variants or amino acid substitutions of a gene on protein function can be measured in a single assay [3, 4]. These assays are of particular clinical interest for genes for which (i) the VUS rate is high (ii) alternative data of high specificity such as case-control data are frequently lacking (iii) reporting is undertaken in the context of a secondary finding and/or (iv) important clinical decision-making is informed, for example risk-reducing surgery in *BRCA1* [5].

The specification of the ACMG framework for individual genes (hereafter termed VCEP gene-specific guidance) has been led by ClinGen Variant Curation Expert Panels (VCEPs), international groups of experts in the relevant group of genes and phenotypes. For *BRCA1, TP53*, and *PTEN*, published MAVEs have been reviewed by the respective VCEP, some of which have been explicitly recommended for use as part of clinical variant classification. Most VCEPs have applied methodology quantifying evidence from functional assays broadly reflecting the Brnich-SVI validation approach [6–8].

However, there remain several challenges around its clinical application as the volume of MAVE data expand. A particular challenge to clinical adoption of new MAVEs is availability of variant truth-sets comprised of positive and negative control variants by which to validate the assays. Previously classified variants in ClinVar are typically used as a variant truth-set, but the number and robustness of ClinVar classifications varies widely among genes [9]. There are many genes for which no VCEP exists, meaning this gene-specific guidance and coordinated variant curation has not been progressed in these cases. Another important challenge is whether there is a single disease associated with variants in a given gene, or whether there is heterogeneity with several different diseases associated to a given gene, often acting through different molecular mechanisms. In this instance it becomes very important that the validity of the MAVE assay for each gene-disease pair is assessed.

Currently, MAVE data are often shared in formats not readily accessible and/or interpretable by clinical users. Datasets are often reported as supplemental data in research papers using a variety of heterogeneous formats. MaveDB has been developed as a platform for deposition of MAVE data, but clinical users are often unaware of its existence and/or unfamiliar with the data format [10]. Further, MaveDB’s interface is currently configured for data scientists seeking full datasets rather than for clinical use cases querying single variants [11]. The accessibility of this data by the clinical community will increase as other platforms (e.g., ClinGen Linked Data Hub, DECIPHER, Ensembl VEP, UCSC) start to import and display the data deposited to MaveDB.

The Atlas of Variant Effects (AVE) Alliance’s Clinical Variant Interpretation (CVI) workstream (https://www.varianteffect.org/workstreams) was established to address these types of challenges and to establish international guidance and resources for standardising variant truth-sets. In addition, there are recent Cancer Research UK-funded initiatives in the UK and National Human Genome Research Institute-funded initiatives in the USA related to clinical validation and dissemination of MAVE data. In parallel, broader issues relating to quantitation and integration of evidence towards clinical variant classification are being evolved via the ACMG and ClinGen groups, with a substantive update to the 2015 ACMG framework due in 2024.

Through this workshop, we sought to bring together the communities developing MAVEs with those clinical-facing communities considering how functional and other types of data should be best used for clinical variant classification, with the aim of debating challenging areas, gathering views on the potential barriers to wider use of MAVE data in clinical variant classification, and identifying future directions which may alleviate these barriers.

## Subjects and methods

### Workshop planning

The 4.5 h workshop—entitled ‘Clinical Application of MAVE Data’—was planned by committee (CT, LS, DA, FR, JF, LB, SF, LM, & SA), including leadership from the AVE Alliance Clinical Variant Interpretation Workstream, and organisational support from Wellcome Connecting Science [12]. Topics and questions were established in advance by the workshop committee.

The workshop agenda was published online at https://coursesandconferences.wellcomeconnectingscience.org/event/curating-the-clinical-genome-20230710/, and was also sent to participants prior to the meeting (Table 1).

**Table 1:** Full Workshop Agenda.

Start	Item	Speaker/Chair
14:00	Registration and Networking	
Session 1: Introductory Talks		
14:20	Welcome and Workshop Overview	Clare Turnbull
14:30	Developing clinical-grade multiplex assays of variant effect	Greg Findlay
14:45	Overview of ACMG variant classification and evidence weighting	Leslie Biesecker
15:00	*BRCA1*, *TP53*, and *PTEN*: what have we learnt so far from correlating MAVE data with clinical observations?	Shawn Fayer
15:20	Q&A and room-wide discussion	Clare Turnbull
Session 2: Correlating Clinical Pathogenicity with Assay Readouts		
15:40	Informal Question Polling	Lea Starita
15:50	On-table discussion questions *(8 tables, 4-6 persons per table)*	
16:10	Refreshment Break	
16:30	Room-wide discussion and formal polling	Lea Starita
Session 3: Disseminating Outputs of MAVEs		
17:00	MaveDB plans, integration between MaveDB and other resources	Alan Rubin
17:10	Informal Question Polling	Frederick Roth
17:20	On-table discussion questions *(8 tables, 4-6 persons per table)*	
17:40	Room-wide discussion and formal polling	Frederick Roth
18:10	Workshop Reflection	

All times are in BST.

### Workshop participants

To encourage representation from both those with expertise in clinical variant classification and those with expertise in development of MAVE assays, the workshop was scheduled between two relevant conferences happening at the Wellcome Sanger Institute—the ‘Curating the Clinical Genome’ (CCG) conference co-hosted by DECIPHER and ClinGen from 10th–12th July, and the ‘Mutational Scanning Symposium’ (MSS) from 13th–14th July organized by the AVE Alliance.

A total of 44 participants attended the workshop, including attendees from nine countries (Table 2). Thirty-one participants were actively invited by the workshop faculty (Table 3) based on their established experience in the field of clinical variant classification or MAVE assay development, and the remaining 13 were selected from applicants who registered interest in the workshop following promotion. Also in attendance were four workshop organisers, who assisted in planning, registration, and note-taking.

**Table 2:** Demographic details of all 44 registered workshop participants.

Affiliation	Total *n*	Breakdown by affiliated country
Academic	22	USA (*n* = 7), UK (*n* = 6), Australia (*n* = 4), Canada (*n* = 1), Democratic Republic of Congo (*n* = 1), Spain (*n* = 1), the Netherlands (*n* = 1), China (*n* = 1)
Clinical	14	USA (*n* = 6), UK (*n* = 5), Germany (*n* = 1), Spain (*n* = 1), Canada (*n* = 1)
Commercial	8	USA (*n* = 6), UK (*n* = 1), the Netherlands (*n* = 1)

Primary affiliation was recorded upon registration, and based on their listed affiliation participants were assigned into the three general categories of ‘Academic’, ‘Clinical’, or ‘Commercial’.

**Table 3:** Workshop faculty details and affiliations.

Faculty Member	Position	Affiliation
Professor Clare Turnbull MD, PhD	NHS Consultant in Clinical Cancer Genetics and Professor in Translational Cancer Genetics	The Institute of Cancer Research, London, UK
Professor Lea Starita, PhD	Associate Professor in Genome Sciences, Co-director of Brotman Baty Advanced Technology Lab	University of Washington, USA
Professor Les Biesecker MD	Chief of the Center for Precision Health Research at the National Human Genome Research Institute (NHGRI)	National Human Genome Research Institute, USA
Professor Frederick Roth, PhD	Professor in Molecular Genetics and Senior Scientist at the Lunenfeld-Tanenbaum Research Institute	University of Toronto, Canada
Dr Greg Findlay MD, PhD	Group Leader of the Genome Function Laboratory	The Francis Crick Institute, UK
Shawn Fayer	Genetic Counsellor graduate student	University of Washington, USA

### Format of workshop

The workshop was separated into three sessions. The first session consisted of introductory talks to provide a baseline level of knowledge and context for the later discussion and was followed by a ‘Question and Answer’ session (Table 1). The second session addressed themes relating to Correlating Clinical Pathogenicity with Assay Readouts (variant truth-sets and quantitative validation). The third session addressed Dissemination of Outputs from MAVEs. The second and third sessions predominantly comprised table discussions, room-wide discussion, informal and formal polling.

### Polling and assembly of response data

Participants were split across eight tables of four to six participants per table. Table seating was determined ahead of time to ensure each table had a combination of participants from clinical, commercial, and academic backgrounds. One facilitator and one note-taker were nominated by each table, where the note-taker recorded key points from discussion, and the facilitator managed discussion and fed back to the room after discussion.

The general format of sessions 2 and 3 were the same – introductory presentation, informal polling, table discussion, room-wide discussion, and formal polling. Poll questions were designed to be of a binary agree/disagree format (Table 4). As such, no poll was conducted for Topic 5 and Topic 7, since these topics were more suited to open discussion and collaborative suggestions than to binary agree/disagree statements.

**Table 4:** Overview of the topics discussed during the workshop, on-table discussion questions, and polled questions.

Topics	Discussion Questions	Poll statement
TOPIC 1: Acceptable model organisms	Should there be a priori guidelines covering which cell types (or model organisms) are acceptable for clinical use, or should we subject assays from every cell type/organism to the same validation standard?	‘Nobody should ever take an assay of a human protein variant expressed in yeast as evidence for clinical variant classification.’
TOPIC 2: Assay-level or variant-level evidence	Using the Brnich-SVI validation approach for assays with a quantitative readout, should all variants receive the same evidence strength as assigned at assay level (grouping), or should every variant receive its own quantitative measure of evidence strength based on a per-variant assay readout (splitting)?	‘Given functional assays that are quantitative, with estimates of experimental error, the strength of evidence derived from a given assay should differ between variants.’
TOPIC 3: Size of Variant truth-sets	What approaches might you take if the set of available clinically-classified missense variants is too small to allow the formal Brnich-SVI validation approach?	‘For a gene without sufficient P/LP variants (e.g., PALB2), the assay can be validated by demonstrating evidence of disease association for functionally-abnormal missense variants as a group in a case-control cohort (in combination with application of nonsense variants as the pathogenic variant truth-set for the Brnich-SVI validation approach).’
TOPIC 4: Validation of missense variants: domain-level or gene-level	In a gene for which there are three recognised ‘functional’ domains, how would you approach validation of pathogenicity/benignity for missense variants? Do you need to do a set of validation variants for each domain, to explore potential for variation in domain-specific effects (eg some might be dominant-negative-type)?	‘MAVE validation should be performed separately for missense variants for each functional domain’
TOPIC 5: Acceptable scope of MAVE data in clinical variant classification	Should it ever be possible to classify a variant as likely pathogenic (or pathogenic) based solely on functional assay results + in silico prediction?	*No poll conducted for this topic*.
TOPIC 6: Standards for dissemination and clinical application of MAVE data	Given that generation of functional data (variant maps) may long predate formal publication, what would be an acceptable venue for initial release of the data to be used for clinical variant classification?	‘MAVE data should only be used in clinical variant classification where it has been validated by the Brnich-SVI validation approach in a published analysis by an accepted authority.
		‘Assays should only be used from manuscripts that have been peer-reviewed as evidence for clinical variant classification’
		‘Journals should only publish papers drawing clinical conclusions from functional assays if those assays have been ‘blessed’ by the relevant VCEP’
		‘MaveDB should not be curated for clinical use. Assay validation for downstream clinical use should be a separate process performed by each end user.’
TOPIC 7: Sharing MAVE data	How should we encourage MAVE data sharing? What are the carrots? What are the sticks?	*No poll conducted for this topic*.
TOPIC 8: Trajectory of the field of genomic variant interpretation	To what extent will delivery of MAVES impact upon and transform clinical variant classification?	‘The term “variant of uncertain significance” will be obsolete by the year 2033.’

Informal polling posed a series of statements to the room, and participants chose if they agreed or disagreed with the statements by holding up green or red cards, respectively. This sparked early discussion, with one to three participants selected from the room to explain why they chose to agree or disagree. Informal polling was followed by table discussions. Each session had four discussion questions, each of which was given to two tables for discussion, therefore each question was discussed by eight to twelve participants in total. After 20 min of discussion, questions were opened to the room, where tables fed back on their key thoughts and all participants were invited to comment. Finally, following opportunity for group discussion of the themes with others, the polls from the start of the session were repeated, with each participant recording on an electronic poll if they agreed or disagreed with the statement.

### Outcomes

Outcomes from the workshop were captured both quantitatively through polling results and qualitatively by the note-takers and the meeting organizers through on-table discussions and in-room feedback.

## Results

### Session 2: Correlating Clinical Pathogenicity with Assay Readouts

The second session focused on five questions relating to MAVE design, variant truth-sets, assay validation and use of MAVE data in clinical variant classification (Table 4).

### TOPIC 1: Acceptable model organisms

MAVEs have been developed to assess the functional effects of variants in a variety of model systems. As an example, yeast assays have been used to assess variants of human orthologs, but these yeast-based assays have been viewed sceptically by many in clinical genetics [13, 14]. Thus, the first topic addressed in table discussions was that of acceptable model organisms, prompted by the question: ‘Should there be a priori guidelines covering which cell types (or model organisms) are acceptable for clinical use, or should we subject assays from every cell type/organism to the same validation standard?’

Within this discussion, it was debated whether there should be a priori predefined types of assays deemed as valid for informing clinical pathogenicity of human disease variants and whether it was necessary to consider the cellular assay under evaluation in the context of the proposed cellular/molecular mechanism underlying clinical pathogenicity. Several participants stated that there should not be prescriptive guidelines regarding cell models for assays, provided that the validation data were robust.

#### Final poll result

35/39 participants *disagreed* with the statement ‘Nobody should ever take an assay of a human protein variant expressed in yeast as evidence for clinical variant classification.’

### TOPIC 2: Assay-level or variant-level evidence

A typical MAVE generates a quantitative score for each variant effect measured in the assay. However, often variants are assigned into binary readouts for the assay based on assay-level thresholds, thus losing the detailed variant-level quantitative information. The table question provided for the topic 2 discussion was ‘Using the Brnich-SVI validation approach for assays with a quantitative readout, should all variants receive the same evidence strength as assigned at assay level (grouping), or should every variant receive its own quantitative measure of evidence strength based on a per-variant assay readout (splitting)?’

Overall, discussion (and the final poll) supported as superior the ‘splitting’ rather than ‘grouping’ of assay results for different variants, on the grounds this reflected consideration of both the robustness of the assay overall and the strength of evidence generated for an individual variant. It was widely recognised that new mathematical approaches and consensus in the methodology would be required if we move to variant-specific evidence-scoring. There was discussion of how hypomorphic variants would be presumed to attain lower variant-specific assay scores, and whether it was appropriate that this might be interpreted as indicating a lesser level of evidence rather than a lower effect magnitude of equivalent evidence strength.

#### Final poll result

All 44 participants polled *agreed* with the statement ‘Given functional assays that are quantitative, with estimates of experimental error, the strength of evidence derived from a given assay should differ between variants.’

### TOPIC 3: Size of variant truth-sets

The OddsPath calculation behind the Brnich-SVI validation approach requires perfect separation of at least 18 known pathogenic and 18 known benign variants to reach ‘strong’ evidence strength. Beyond a handful of genes, missense variants of known clinical effect just don’t exist in the numbers required for assay validation to reach ‘strong’ and some don’t have enough variants to even attempt the Brnich-SVI validation approach. This quandary prompted the question: ‘What approaches might you take if the set of available clinically-classified missense variants is too small to allow the formal Brnich-SVI validation approach?’

It was recognised that case-control data provided powerful evidence of pathogenicity, but was of limited availability for most gene-phenotype dyads and where available was typically of limited signal for most variants due to low variant frequencies even in sizeable datasets. It was widely agreed that collapsed (burden) case-control signal for a pre-specified group of variants functionally-abnormal on an assay provided a ‘standard-candle’ by which the performance of the assay for missense variants was demonstrated at ‘summary-level’ for scenarios for which there is a paucity of individually-classified variants. In conjunction with this approach, where missense variants were recognised to act by loss-of-function, nonsense variants might be used for Brnich-SVI quantitative validation of the assay. It was suggested that only through this type of approach would it be possible to advance the current ‘Catch 22’ for genes such as *PALB2* and provide functional evidence that could contribute evidence of pathogenicity for some individual rare missense variants (which variant classifications would then in due course be available for clinical validation of subsequent assays).

It was proposed that to advance the size and availability of benign variant truth-sets, there might be utility in systematically identifying groups of variants based on frequency within population databases such as gnomAD [15]. It was suggested that these ‘proxy-benign’ variant truth-sets could be further improved with systematic addition of in silico scores (or, where available, case-control data). Participants highlighted that often an individual clinical ClinVar classification of ‘benign’ is based on equivalent evidence.

#### Final poll result

38**/**42 polled participants *agreed* with the statement: ‘For a gene without sufficient P/LP variants (e.g., *PALB2*), the assay can be validated by demonstrating evidence of disease association for functionally-abnormal missense variants as a group in a case-control cohort (in combination with application of nonsense variants as the pathogenic variant truth-set for the Brnich-SVI validation approach).’

### TOPIC 4: Validation of missense variants: domain-level or gene-level

The fourth topic addressed was whether missense variants should be validated at the gene level or require validation at domain level (on the presumption of heterogeneity of mechanism of pathogenicity). The table question provided for discussion was ‘In a gene for which there are three recognised ‘functional’ domains, how would you approach validation of pathogenicity/benignity for missense variants? Do you need to do a set of validation variants for each domain, to explore potential for variation in domain-specific effects (e.g., some might be dominant-negative-type)?’

Within this topic, it was debated how a single assay may fail to accurately capture both missense (or other non-truncating non-synonymous) variants acting via loss-of-function and other variants acting by gain-of-function (dominant negative) mechanisms. If using a single assay for that gene quantifying loss-of-function, an assumption is therefore being applied that all missense variants throughout the gene are acting by an identical loss-of-function mechanism to the selected missense pathogenic variant truth-set. It was therefore debated whether missense variants should be validated not at gene level but at domain level. Most participants agreed this to be a valid issue. However, significant concern and weariness were expressed at the implications of requiring missense variant truth-sets for each domain in the gene, given the substantial challenges already in identifying sufficient missense variants for variant truth-sets at gene level, which was reflected at polling. Furthermore, there was concern raised regarding how domains would be defined and whether variation in mechanisms of pathogenicity would even be distributed by contiguous linear domains. Single-cell transcriptomic studies were proposed as a means of validating variants for each domain.

#### Final poll result

25/41 polled participants *agreed* with the statement ‘MAVE validation should be performed separately for missense variants for each functional domain.’

### TOPIC 5: Acceptable scope of MAVE data in clinical variant classification

According to the original ACMG guidance, functional evidence alone would not be sufficient to reach likely pathogenic [1]. Updated guidance from ClinGen would technically allow functional data to reach “very strong” which would be enough for a likely pathogenic classification without additional sources of evidence. Furthermore, when the ACMG guidance was written in silico evidence was capped at the supporting level. Improvements in machine learning have vastly increased the accuracy of in silico predictors and the ClinGen Sequence Variant Interpretation working group has issued updated guidance such that some in silico predictions can be applied to variant classification at strong evidence [16]. This suggests that, for many variants, functional data plus an in silico prediction could equate to a likely pathogenic classification without any other input. This prompted the question ‘Should it ever be possible to classify a variant as likely pathogenic (or pathogenic) based solely on functional assay results, if it were the only available data on a variant?’ and discussion on classifications based solely on combination of functional results and in silico predictions.

It was debated whether functional data in conjunction with in silico and population frequency data (i.e., absence in controls) would be sufficient. It was recognised this would be an increasing issue in classification of variants observed in the context of secondary findings, population testing, or very weak clinical indications. Some participants advocated for a purely quantitative approach, by which an assay score attaining a sufficiently high likelihood ratio would be sufficient for the variant to be classified as (likely) pathogenic. Others were uncomfortable that a variant could be classified as (likely) pathogenic without ever being reported in an individual with the appropriate phenotype. A middle-ground was proposed as an option, where variants could be classified by the laboratory based on functional and/or in silico data, which could then be interpreted by the clinician using patient-specific data to provide the final classification.

It was recognised that this question related in part to the standards by which the assay had been clinically validated, in particular, the degree to which the variant truth-sets used for validation were based on robust classifications using robust phenotypic data. It was also debated in this context the role of ‘an accepted clinical authority’ such as a VCEP in taking responsibility for the clinical dataset used for validation of an assay.

### Session 3: Dissemination of Outputs from MAVEs

MAVE data will only be useful clinically if it is disseminated according to FAIR (Findability, Accessibility, Interoperability, and Reusability) standards and with the transparency required to assess its accuracy as defined by Wilkinson et al. in 2016 [17]. The final workshop session shifted the focus onto themes relating to how MAVE data should be disseminated, when should MAVE data be considered ready for use, and how data-sharing might be incentivised (Table 4). The session finished with a discussion of the trajectory of the field of clinical variant classifications and VUS.

### TOPIC 6: Standards for dissemination and clinical application of MAVE data

As in many other scientific fields, MAVE data may go through many stages of public release. The data may be shared as part of a consortium data set, uploaded to a preprint server or published as part of a peer reviewed study [12, 18]. The need to better understand when the data is ready for clinical use prompted the sixth question: ‘Given that generation of functional data (variant maps) may long predate formal publication, what would be an acceptable venue for initial release of the data to be used for clinical variant classification?’

Within this topic, it was debated how MAVE data should be made available to the clinical user community, by whom it should be ‘approved’ and in particular whether/how/where these data might be made available prior to formal publication. Participants considered several options for pre-publication data release, including bioRxiv/medRxiv publication, release onto MaveDB or upload to ClinVar. There were mixed opinions as to how the clinical community would perceive data available from these sources, but also recognition that peer review and formal publication did not necessarily in itself reflect attainment of a standard of (clinical) validation of the assay: ‘A lot of data in scientific databases would then be unusable as the data have not been published in a journal’, whilst other data could have been ‘…validated robustly but not peer reviewed’. Participants were broadly supportive of submission to bioRxiv/medRxiv and of using MaveDB as a repository for making MAVE data available in the interim ahead of publication.

Also discussed was the role of MaveDB for sharing MAVE data with the clinical community. It was also highlighted the value of MaveDB in retaining a venue for upload of MAVE data with ease of access across laboratories and countries. However, there were disparate views as to what level of clinical review/validation was required upstream of release, and what would be minimal standards for associated metadata released at assay-level and at variant level to assert technical quality and validation against variant truth-sets.

Overall, it was felt that the most important form of clinical validation was a formal Brnich-SVI validation undertaken by an authoritative expert clinical diagnostic body such as a VCEP group. However, there was varying opinion as to the extent to which Brnich-SVI style scoring should be essential before any data release. There was also mixed opinion as to importance of this review being by formally designated VCEP, versus by other relevant clinical experts. It was mentioned for example, that for some genes there is no VCEP and ‘there is not enough bandwidth or resource to generate and form more VCEPs’. It was also mentioned that MAVE generation and other relevant data in the literature ‘…move faster than VCEPs update their recommendations’.

There was lengthy consideration of assay-level and variant-level metadata that might be important to present in MaveDB, relating to both technical validity and clinical validation. Participants discussed the merits of different options for presenting assay validation in MaveDB, including (i) illustration of separation of synonymous and nonsense variants, (ii) concordance with a pre-defined variant reference set or (iii) evaluation by an external authority (e.g., a VCEP).

Some participants advocated for the value of a central ‘validation service’ associated with MaveDB, by which newly-released MAVEs could be reviewed and quantitatively clinically validated. Whilst it was widely agreed that this would provider consistency, reduce redundancy and expedite clinical availability of the data, there was no clear proposal how and by whom this service might be delivered. There was also discussion regarding providing pre-defined variant truth-sets in MaveDB for each gene.

Such a resource was widely recognised to be advantageous versus use of disparate variant truth-sets by different MAVE submitters, which potentially may be small, include variants of low ‘truth’, and undermine comparability of performance between MAVE datasets. Again, whilst maintaining pre-defined variant truth-sets within MaveDB was widely agreed to be of high value, it was unclear how and by whom this activity would be resourced and maintained.

#### Final poll results

35/41 participants *disagreed* with the statement ‘MAVE data should only be used in clinical variant classification where it has been validated by the Brnich-SVI validation approach in a published analysis by an accepted authority.’

Twenty-nine of thirty-nine participants *disagreed* with the statement ‘Assays should only be used from manuscripts that have been peer-reviewed as evidence for clinical variant classification’.

Thirty-seven of thirty-nine participants *disagreed* with the statement ‘Journals should only publish papers drawing clinical conclusions from functional assays if those assays have been ‘blessed’ by the relevant VCEP’.

Twenty-three of thirty-six participants *disagreed* with the statement ‘MaveDB should not be curated for clinical use. Assay validation for downstream clinical use should be a separate process performed by each end user.’

### TOPIC 7: Improving sharing of MAVE data

The seventh topic addressed in table discussions was how to improve and incentivise sharing of MAVE data, with the table question provided for discussion: ‘How should we encourage MAVE data sharing? What are the carrots? What are the sticks?’.

Incentives and mechanisms proposed included (i) improved visibility and recognition of investigator data in MaveDB, (ii) tracking MAVE data references in publication and contacting investigators, (iii) informing researchers when their MAVE data was used to resolve patient variants, (iv) collaboration between VCEPs and MaveDB, (v) requirement for data submission to MaveDB prior to journal submission, and (vi) requirement for data sharing plans in MAVE grant submissions. The value of improving the visibility of MaveDB was discussed, with cross-referencing to ClinVar offered as a potential option.

### TOPIC 8: Trajectory of the field of genomic variant interpretation

We closed the meeting with a final discussion of the question: ‘To what extent will delivery of MAVEs impact upon and transform clinical variant classification?’

Some participants articulated high expectations that MAVEs would dramatically transform the classification of rare variants and that VUS may be ‘defined out of existence by giving more quantitative scores to criteria’, or by using evidence combinations. One of those less optimistic highlighted that, in circular fashion, lack of variant truth-sets was a hurdle to delivering impactful clinical classifications from MAVEs, with another stating that ‘There are more clinical disease genes being assigned all the time, which won’t have key evidence ready for classification [of truth-set variants]’. One participant also highlighted that ‘the bulk of the genome is non-coding, … and so there is no way to apply most evidence (meaning these will end up as VUS)’.

#### Final poll result

34/39 participants *disagreed* with statement: ‘The term ‘variant of uncertain significance” will be obsolete by the year 2033’.

## Discussion

### Correlating Clinical Pathogenicity with Assay Readouts

The workshop articulated the urgent requirement for more systematic clinical validation of assay data. However, clear solutions were lacking for how this clinical validation service might feasibly be delivered as data curation efforts are labour intensive and difficult to fund through many grant mechanisms. The challenges in generating high-quality, well-sized variant truth-sets for assay validation were repeatedly raised as a fundamental barrier. Reflecting this issue, there was widespread consensus regarding the need for more flexible approaches to assay validation, such as aggregating sets of rare missense variants with similar assay results to validate which assay outcomes correspond best with clinical case-control data, and more flexible approaches to the systematic generation of benign truth-sets. However, work will need to be done to understand how to weight functional data validated by case-control studies. Another drawback of using case-control data is the lack of clinical cohorts for most gene-disease pairs; however, large biobanks of sequenced individuals linked to health records such as UK Biobank and All of Us should provide an increasingly well-powered platform for performing case-control studies moving forward [19, 20].

The current Brnich-SVI validation approach categorizes functional evidence based on validation of the MAVE assay as a whole: there was unanimous consensus regarding moving to variant-specific scores. An example of such ‘splitting’ and assignation of variant-specific scores from MAVE data has previously been achieved by calibration of robust predictive models for pathogenic and benign variants, and could theoretically be applied in-kind for other MAVEs, proving the feasibility of generating variant-specific scores provided appropriate analyses are conducted [21]. Hypomorphic variants were agreed to be a substantial challenge in the short-term with regard to validation, but one in which MAVEs might ultimately assist [22, 23].

There was recognition regarding the complexity by which missense variants may confer pathogenicity, with over half of participants concurring that separate validation should be undertaken for missense variants for each functional domain. This is an area requiring substantial future attention to ensure that the assessed function of genes and domains is relevant in a disease-context, particularly where genes encompass multiple functions and domains confer different functions, and the clinical validity of each gene-disease pair should be robustly investigated prior to the MAVE.

### Dissemination of Outputs from MAVEs

Opinions differed quite widely as to the extent and mechanisms by which MAVE data might be ‘signed-off’ or ‘approved’ for use in clinical variant classification. Most participants agreed that peer-review and formal publication in a journal was neither necessary nor sufficient as a mechanism by which MAVE data is reviewed ahead of clinical application, with specific concern regarding publication lag and lack of detailed data scrutiny within standard peer review. Most participants did not deem that sign-off by a designated VCEP was essential ahead of clinical application of MAVE data. There was also debate regarding how and whether data in MaveDB might best be made accessible to clinical users, both in terms of assurances of technical and clinical validity but also its clarity of presentation. The value of MaveDB as a universal hub for MAVE data was widely agreed, enabling researchers to identify concurrent work on the same gene, cross-comparison of outputs, and a single repository for the user-community. How best to encourage data submission was debated, with collaboration, visibility and an audit trail of MAVE data cited as the main incentives.

### Limitations

The constituency of the group was determined by attendance at the preceding/following research conferences. Heterogeneity regarding pre-meeting expertise was mitigated by the introductory talks, although not all questions received responses from all participants (responses ranged 36–44). Polling was via binary statements, meaning nuanced participant views were not reflected. Consensus was not attained for most discussion questions; the aim of the workshop was discussion and broad gauging of opinion rather than generation of recommendations. Further workshops/meetings will be required to develop suggestions into actionable work to progress translation of MAVE results towards utility in clinical variant classification.

### Future directions

The following are potential actions based on the suggestions from this workshop:

- Focused consensus workshop(s) with relevant selected groups to further develop suggestions around clinical use of MAVE data.
- A specific MAVE Evaluation Working Group to develop validation standards for MAVEs (including development of variant truth-sets).
- Specific software and user interface changes to MaveDB to support the community (e.g., DOIs for submitted data).
- Establishment of cross-working with ClinVar/ClinGen to incorporate links to MaveDB and optimise clinical utility of the platform.

## Conclusion

As the field of MAVE development progresses, there will be increasing generation of assay data that can and will be used in clinical variant classification. The workshop was convened in recognition of the urgent need to systematise and standardise clinical validation of these MAVE data, and to make both the MAVE and validation data available to clinical and research communities, and made fruitful initial headway in exploring approaches and challenges therein. Future directions include more focused additional workshops and the creation of working groups.

## Data Availability

All data generated or analysed during this study are included in this published article.
